# Effect of a Multistrain Probiotic on Feline Gut Health through the Fecal Microbiota and Its Metabolite SCFAs

**DOI:** 10.3390/metabo13020228

**Published:** 2023-02-03

**Authors:** Yifei Li, Ilyas Ali, Zhiqi Lei, Yanan Li, Min Yang, Caixia Yang, Lian Li

**Affiliations:** 1 College of Animal Science and Technology, Nanjing Agricultural University, Nanjing 210095, China; 2 Department of Medical Cell Biology and Genetics, Health Science Center, Shenzhen University, Shenzhen 518060, China

**Keywords:** *Saccharomyces boulardii*, *Pediococcus acidilactici*, gut microbiota, SCFAs, cat, gut health

## Abstract

With the increasing awareness of raising pets following scientific methods, people are becoming increasingly more interested in the nutrition and health of pets, especially their intestinal health, which has become a research hotspot. Both *Saccharomyces boulardii* and *Pediococcus acidilactici* are probiotics with strong probiotic properties that can maintain the balance of intestinal flora. However, the role of *Saccharomyces boulardii* and *Pediococcus acidilactici* in felines has not been comprehensively studied to date. The aim of this study is to investigate the effect of multistrain probiotics consisting of *Saccharomyces boulardii* and *Pediococcus acidilactici* on the gut health of felines by modulating gut microbes and the production of metabolite SCFAs. The results show that the multistrain probiotic did not alter the intestinal microbial diversity and structure of short-haired domestic cats, promoted the colonization of beneficial bacteria, increased the levels of microbiota-derived SCFAs and fecal antioxidants, and reduced the levels of fecal inflammatory markers. In conclusion, the use of a multistrain probiotic in healthy, short-haired domestic cats can promote gut health by modulating gut microbes, improving microbiota-derived SCFA production, reducing inflammatory conditions, and improving antioxidant status. These results provide new insights for further exploration of the role of probiotics in the gut microbiome of cats.

## 1. Introduction

Pet nutrition and health are attracting increased attention in the literature, as pets play a greater role in the daily lives of human beings. The gastrointestinal microbiome of pets is closely related to pet health. Gut microbes, which are typically categorized as commensal, pathogenic, or opportunistic bacteria [1], are observed in the animal’s digestive tract, and each of them has its own distinct flora balance. Intestinal microorganisms participate in the digestion, absorption, and metabolism of nutrients in the host body and play an important regulatory role in the metabolism of nutrients, such as lipid metabolism, carbohydrate metabolism, protein metabolism, and vitamin synthesis. The composition and status of the gut microbiome are closely associated with the intestinal health of the host, and the imbalance of the host intestinal microbiome may lead to a series of disorders in the body, such as gastrointestinal diseases, metabolic diseases, obesity, and immune system diseases.

Probiotics are defined as “live microorganisms that are beneficial to the host when ingested in sufficient amounts” [2], and the most commonly studied in the literature are *Lactobacillus*, *Bifidobacterium*, and yeasts [3]. Probiotics are capable of lowering the pH value, creating an acidic environment, reducing the reproduction of harmful microorganisms, increasing the number of beneficial bacteria, interacting directly with immune cells [4], and participating in regulating the immune balance of digestive tract flora, thereby contributing to the prevention and treatment of gastrointestinal-related diseases. Studies have proven that probiotics are effective in the treatment of acute infectious diarrhea, antibiotic-associated diarrhea, *Clostridium difficile*-associated diarrhea, hepatic encephalopathy, ulcerative colitis, irritable bowel syndrome, functional gastrointestinal disorders, and necrotizing enterocolitis [5,6,7,8,9].

The function of probiotics is closely related to the species of microorganisms colonized in the intestines. The interaction occurring among probiotics, host cells, and intestinal flora is a key factor affecting host health. Probiotics can regulate intestinal mucosal immunity, interact with symbiotic bacteria or potentially harmful pathogens, and produce metabolites, such as short-chain fatty acids (SCFAs), which help to inhibit and eliminate potential pathogens, thereby improving the intestinal microbial environment, strengthening the intestinal barrier, and reducing inflammation, with the purpose of enhancing antigen-specific immune responses [10]. An aqueous probiotic suspension was studied in in vitro human intestinal models, and it was observed that probiotics could increase the production of SCFAs and anti-inflammatory cytokines [11]. Studies using *Lactobacillus paracasei* SSP in d-galactose-induced aging mice determined that probiotics improved memory and learning ability in mice by increasing SCFA production and inhibiting cell apoptosis and brain injury [12]. The growth of bacteria in microorganisms affects the type and amount of fermentation products. After sugar is phosphorylated, bacteria can be converted to pyruvate or pyruvate and additional acetyl phosphates by glycolysis (the Embden–Meyerhof–Parnassian pathway), the Enter–Doudoroff pathway, or the *Bifidobacterium* pathway [13].

*Saccharomyces boulardii* is the only yeast among intestinal probiotics. Unlike other Saccharomyces cerevisiae strains, *Saccharomyces boulardii* is physiologically and metabolically tolerant to low pH levels and resistant to the gastric environment [14]. *Saccharomyces boulardii* exhibits distinct probiotic properties, including improved intestinal barrier function, competitive exclusion of pathogens, production of antimicrobial peptides, and immunomodulatory and nutritional effects [15]. *Saccharomyces boulardii* significantly ameliorates colon damage and regulates inflammatory responses in mice with colitis induced by dextran sulfate sodium (DSS) through changes in the microbiome composition and SCFA metabolism [16]. *Pediococcus acidilactici* is a facultative anaerobic bacterium of Firmicutes with a high salt tolerance, and it plays an important role in enhancing immunity, producing bacteriocins, maintaining the balance of intestinal flora, and improving the intestinal barrier [17]. The intervention of *Pediococcus acidilactici* FZU106 was observed to obviously increase SCFA levels in high-fat diet (HFD)-induced hyperlipidemic rats, which was strongly associated with changes in gut microbial composition and metabolism [18]. *Saccharomyces boulardii* has been observed to be safe for use in dogs with chronic enteritis and to control clinical symptoms better than standard treatment alone [19]. The use of *Enterococcus faecium* SF68 in cats infected with latent feline herpesvirus 1 (FHV-1) reduced morbidity and maintained fecal microbiota diversity [20]. Studies on *Saccharomyces boulardii* and *Pediococcus acidilactici* in pet cats have rarely been reported, and this trial investigated the effect of a multistrain probiotic preparation added to the diet on intestinal fecal microbial diversity, microbiota-derived SCFA production, antioxidative status, and fecal inflammatory markers in healthy short-haired domestic cats. This study provides an innovative platform to further investigate the significance of *Saccharomyces boulardii* and *Pediococcus acidilactici* with respect to the intestinal health of companion animals.

## 2. Materials and Methods

### 2.1. Study Probiotic

The multistrain probiotic preparation contained approximately 2.0 × 10^10^ CFU/g of *Saccharomyces boulardii* and approximately 2.5 × 10^10^ CFU/g of *Pediococcus acidilactici* mixed in a 1:1 ratio. *Saccharomyces boulardii* was supplied by the Lallemand Animal Nutrition Group and is the strain used in Levucell SB^®^. *Pediococcus acidilactici* (Mito 5051) was provided by Imagilin Technology LLC (Frederick, MD, USA). In the test, both the multistrain probiotic preparation and a basic diet were provided by Nanjing Xintengrui Biotechnology Co., Ltd. (Nanjing, China).

### 2.2. Animals

A total of 12 short-haired domestic cats between the ages of 2 and 4 years with an average body condition score of 3.5 points (on a 5-point scale) were used in this study. On day 10 of the trial, 1 of the 12 cats in the control group (male) suffered trauma and withdrew from the study; additionally, a cat (male) in the probiotic group dropped out of the study due to a skin disease that developed during the trial. Therefore, 10 cats (4 males and 6 females) were fully involved in the entire trial. They were randomly divided into control and probiotic groups, each containing two male and three female cats (*n* = 5). Animal experiments were approved by the Laboratory Animal Welfare Ethics Review Committee of Nanjing Agricultural University.

### 2.3. Experimental Design

A total of 12 short-haired domestic cats were fed with a cat basal diet and acclimatized for 1 week with free access to water. After 1 week of acclimatized feeding, the cats were randomly divided into 2 groups of 6 replicates each, with 1 cat per replicate. The control group was fed a basic diet, and the probiotic group was fed a multistrain probiotic preparation on the basis of the control group. The dosage of probiotics was added according to the cat’s weight (0.5 g/kg).

### 2.4. Sample Collection and Fecal Microbiota Analysis

Fresh fecal specimens obtained from all subjects were collected on day 28. According to previously described methods [21], DNA extraction and sequencing were completed by Shanghai Biozeron Biotechnology Co., Ltd. (Shanghai, China). The V3–V4 region of the bacterial 16S rRNA gene was amplified by PCR using primers 341F and 806R. The purification of amplification products was performed using an AxyPrepDNA gel extraction kit (Axygen Biosciences, Union City, CA, USA). Based on the preliminary results of electrophoresis, PCR products were detected and quantified with a QuantiFluor-ST™ Blue Fluor Quantitative System (Promega Corporation, Madison, WI, USA). Isomolar pooled and paired-end sequences of purified amplification products on the Illumina platform (2 × 250) were obtained according to the procedure described in [22].

### 2.5. Fermentation Metabolite Analysis

Concentrations of short-chain fatty acids (SCFAs) were determined by gas chromatography according to the method described in a previous study [23].

### 2.6. Enzyme-Linked Immunosorbent Assay (ELISA) for Fecal Inflammatory Markers

After mixing feces with normal saline in equal amounts, fecal supernatant samples were collected for further analysis. The concentration of inflammatory markers, such as fecal calprotectin (FC), lactoferrin (LF), matrix metalloproteinase-9 (MMP-9), and myeloperoxidase (MPO), in the supernatant were determined by an enzyme-linked immunosorbent assay (ELISA); then, the assay procedure was performed using a cat CALP ELISA kit (ANG-E71001C), cat LTF ELSA kit (ANG-E71005C), cat MMP-9 ELISA kit (ANG-E71013C), and cat MPO ELISA kit (ANG-E71014C) according to manufacturer’s instructions (Nanjing Jiancheng Bioengineering Institute, Nanjing, China).

### 2.7. Fecal Supernatant Antioxidant Capacity Analysis

The contents of glutathione (GSH), superoxide dismutase (SOD), and malondialdehyde (MDA) in fecal supernatants on day 28 were determined using GSH (A006), SOD (A001), and MDA (A003) commercial assay kits, respectively (Nanjing Jiancheng Bioengineering Institute, Nanjing, China).

### 2.8. Statistical Analysis

The data were analyzed using SPSS 23.0 statistical software with an independent *t*-test and expressed as mean ± standard deviation (SD). The difference between the two means was considered statistically significant when *p* < 0.05. Data visualization was performed using GraphPad Prism version 8.0.2.

## 3. Results

### 3.1. Analysis of Intestinal Flora Structure of Healthy Cats

**Figure 1:**
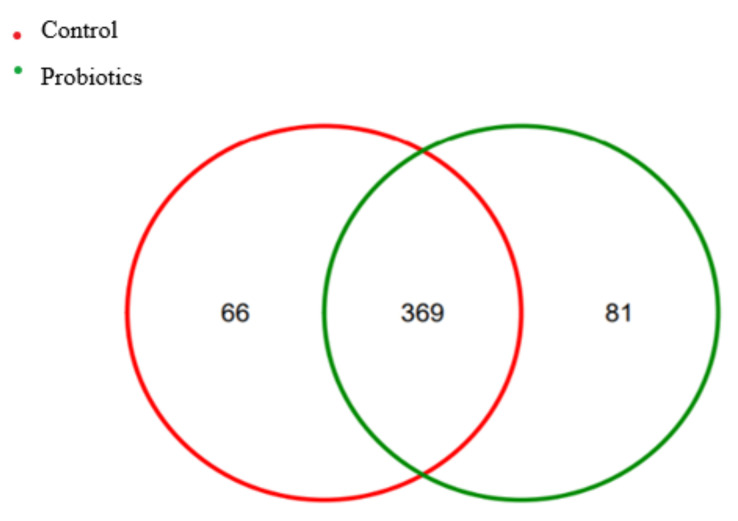
OTU Venn diagram.

As shown in Figure 1, the number of OTUs in the control group was 435, and the number of OTUs in the probiotic group was 450, with a total of 369 OTUs in common between the two groups. The number of OTUs in the probiotic group was 81, compared to 66 in the control group alone.

### 3.2. Phylum-Level Structural Analysis

**Figure 2:**
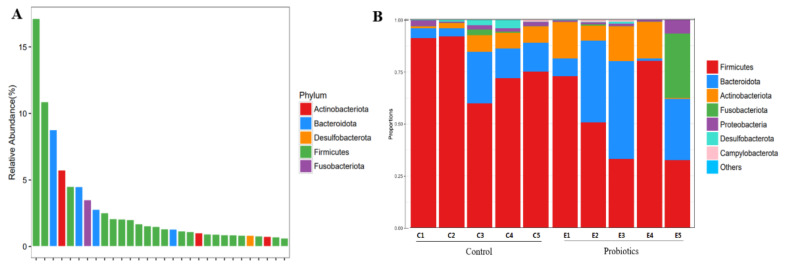
Phylum-level structural analysis. (**A**) The 30 most dominant species in all samples. (**B**) Relative abundance of the gut microbiota at the phylum level.

A few species (genus level) with the highest sampling frequency (the 30 most dominant species) were selected, and the phylum taxonomic information corresponding to the species was displayed; species with the same color indicating their origin from the same phylum were also displayed. The results show that there were five dominant phyla (Figure 2A), namely Actinobacteriota, Bacteroidota, Desulfobacterota, Firmicutes, and Fusobacteriota.

The results show (Figure 2B) that the control and probiotic groups were mainly composed of Actinobacteriota, Bacteroidota, and Firmicutes, whereas the control group was dominated by Firmicutes. The phylum Firmicutes had the highest relative phylum-level frequency in the probiotic group, followed by the phylum Bacteroidota. Table 1 shows that there was no significant difference in the relative abundance of microorganisms at the phylum level between the control and probiotic groups (*p* > 0.05).

### 3.3. Genus-Level Structural Analysis

**Figure 3:**
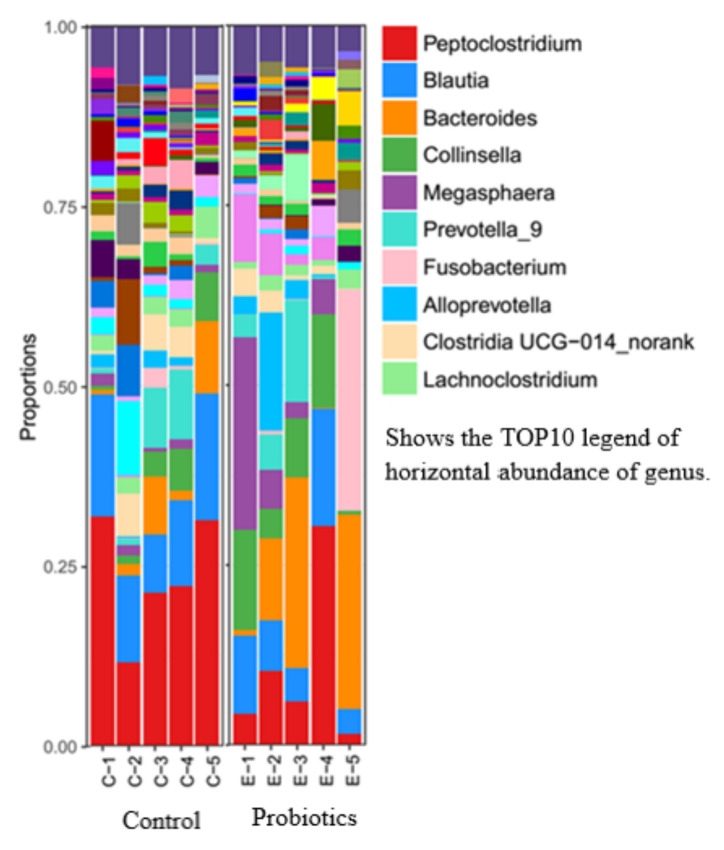
Relative abundance of the gut microbiota at the genus level.

At the genus level (Figure 3), the microbes with a high abundance in the control group were Peptoclostridium and Blautia, whereas the content of Bacteroides was higher in the probiotic group. There was no significant difference in the relative abundance of the top-10 microbial species between the control and probiotic groups (*p* > 0.05).

### 3.4. Alpha Diversity Analysis

The Chao index indicates species abundance. The ACE, Shannon, and Simpson indices are used to calculate species richness, and the evenness index indicates the homogeneity of the microbial community. As shown in Table 2, there were no significant differences (*p* > 0.05).

### 3.5. Dimension Reduction Analysis

**Figure 4:**
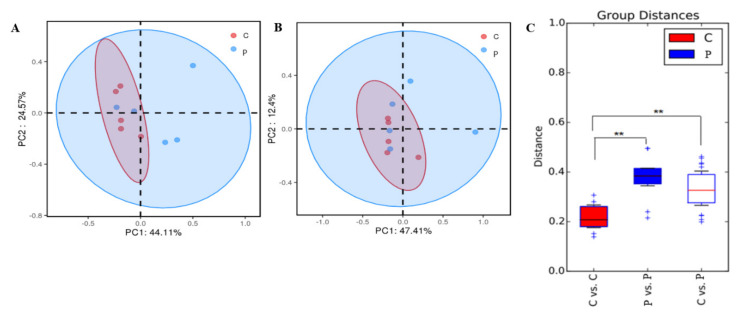
Dimension reduction analysis. (**A**) Principal coordinate analysis (PCoA) of weighted UniFrac distances of 16S rRNA genes. (**B**) Principal coordinate analysis (PCoA) of unweighted UniFrac distances of 16S rRNA genes. (**C**) UniFrac distance matrix plot. The pink circles represent the control group, while the blue circles represent the probiotic group. **: the difference between the control and probiotic groups was extremely significant (*p* < 0.01).

Principal coordinate analysis (PCoA) is based on sample information reflected in the form of points on a two-dimensional plane. Small sample intervals indicate species with similar compositional structures; therefore, samples with a high community structure similarity tend to cluster together, whereas samples with considerable community differences are placed far apart. In Figure 4A,B, the PCoA analysis shows that there was no significant separation between fecal samples of the control and probiotic groups (ANOSIM similarity analysis, weighted UniFrac distance *p* = 0.052, and unweighted UniFrac distance *p* = 0.265).

The weighted UniFrac distance reflects the similarity of gut microbial communities in terms of phylogenetic tree overlap. The greater the distance, the greater the species and abundance of microbial communities between the two groups. As shown in Figure 4C, the weighted UniFrac distances between cats in the probiotic group were greater than those between the control groups, and the difference was highly significant (*p* < 0.01).

### 3.6. LEFSe Difference Analysis

**Figure 5:**
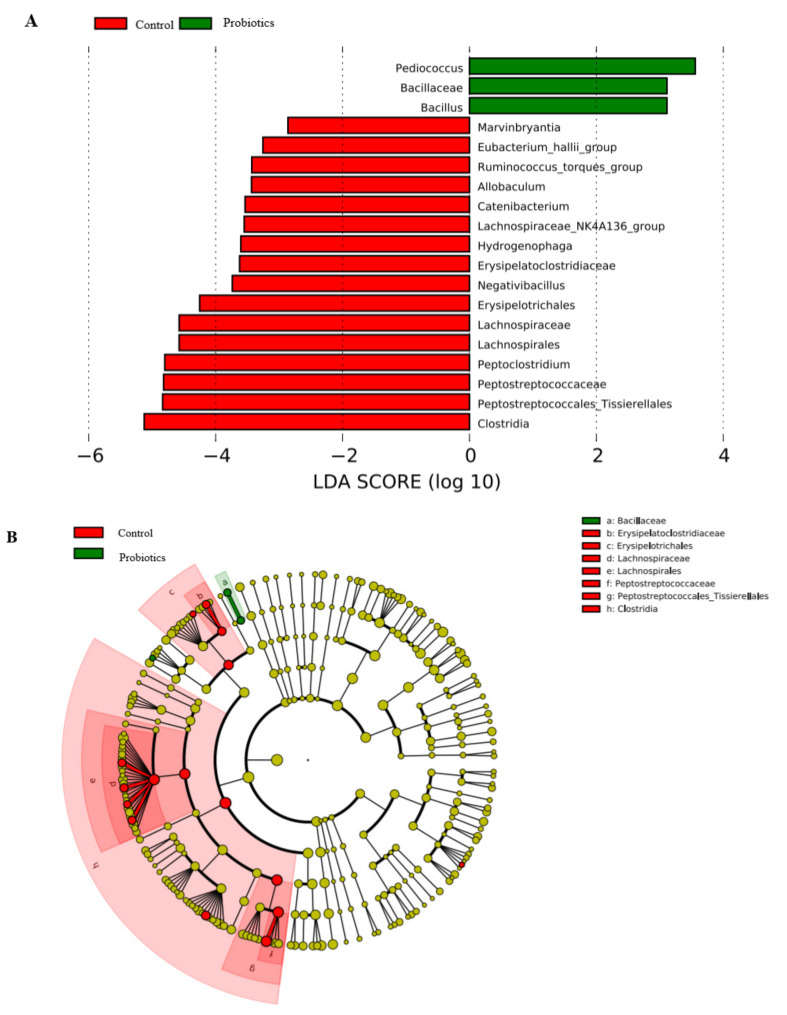
LEFSe difference analysis. (**A**) LDA scores for microbial taxa with significant roles in both groups. (**B**) 16S rRNA sequencing analysis of differences in functional gene abundance in the guts of cats in the two groups.

Figure 5A presents species with significant differences in abundance between groups. The differences are represented by LDA (linear discriminant analysis) scores, and the histogram length represents the contribution of different species (LDA score). Figure 5A shows that 16 species of Firmicutes and Proteobacteria play an important role in the control group. These 16 groups of bacteria are mainly *Bacillus* and *Clostridium* of the phylum Firmicutes and Proteobacteria. The three groups of bacteria that play an important role in the probiotic group are *Pediococcus*, Bacillaceae, and *Bacillus* of Firmicutes.

Furthermore, Figure 5B shows the clade of different species, with circles radiating from the inside to the outside representing the taxonomic levels ranging from phylum to genus (or species). The red nodes indicate microbial taxa that play an important role in the control group, and green nodes indicate microbial taxa that play an important role in the probiotic group. Microbiota that played an important role in the control group were Erysipelatoclostridiaceae, Erysipelotrichales, Lachnospiraceae, Lachnospirales, Peptostreptococcaceae, Peptostreptococcales_Tissierellales, and Clostridia. An important player in the probiotic group is Bacillaceae.

### 3.7. Microbiota-Derived SCFA Production

**Figure 6:**
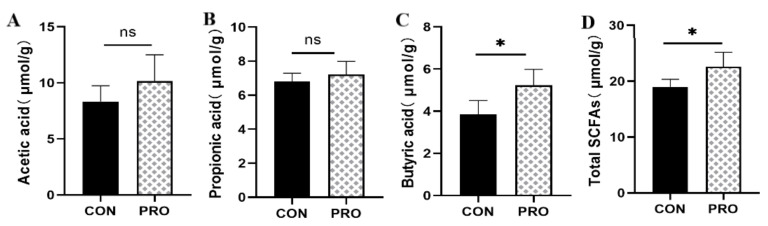
Effect of a multistrain probiotic preparation on fecal fermentation metabolites in healthy short-haired domestic cats. (**A**–**D**) Concentrations of acetic acid (**A**), propionic acid (**B**), butyric acid (**C**), and total SCFAs (**D**) detected by gas chromatography. Data are presented as mean ± SD. * *p* < 0.05; ns, *p* > 0.05 compared with the control group.

The effect of a multistrain probiotic on fecal fermentation metabolites is presented in Figure 6. Both fecal butyric acid and total SCFA concentrations were elevated in the probiotic group compared to the control group (*p* < 0.05). There were no differences in acetic and propionic acid quantities as compared to the control group.

### 3.8. Fecal-Related Inflammatory Indicators

**Figure 7:**
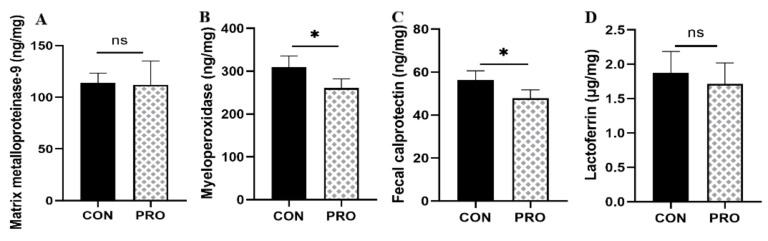
Effect of a multistrain probiotic preparation on inflammatory markers in feces in healthy short-haired domestic cats. (**A**–**D**) Concentrations of matrix metalloproteinase-9 (**A**), myeloperoxidase (**B**), calprotectin (**C**), and lactoferrin (**D**) in feces. Data are presented as mean ± SD. * *p* < 0.05; ns, *p* > 0.05 compared with the control group.

Concentrations of inflammatory indicators in cat feces at each stage were determined using ELISA assay kits. As shown in Figure 7, the probiotic group presented less myeloperoxidase (MPO) and fecal calprotectin (FC) activities on day 28 than the control group (*p* < 0.05).

### 3.9. Fecal Antioxidant Capacity

**Figure 8:**
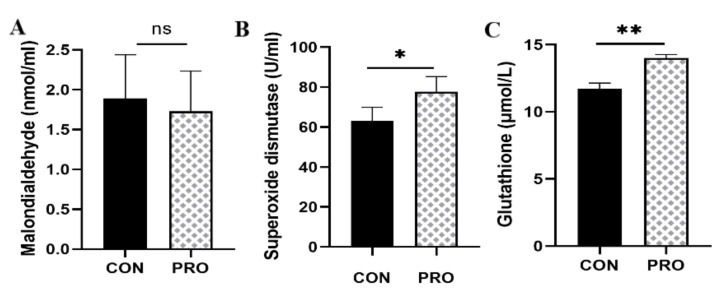
Effect of a multistrain probiotic preparation on fecal antioxidant capacity in healthy short-haired domestic cats. (**A**–**C**) Concentrations of malondialdehyde (**A**), superoxide dismutase (**B**), and glutathione (**C**) detected by commercial assay kits. Data are presented as mean ± SD. * *p* < 0.05; ** *p* < 0.01; ns, *p* > 0.05 compared with the control group.

As can be observed in Figure 8, compared with the control group, the probiotic group presented higher (*p* < 0.05) superoxide dismutase (SOD) and (*p* < 0.01) glutathione (GSH) activity. There was no significant difference in malondialdehyde (MDA) activity between the control and probiotic groups.

## 4. Discussion

Despite the fact that probiotics can improve host health by modulating the gut microbiota, typically through metabolic interactions mediated by stimulation of resident bacteria to alter the abundance of pathogenic bacteria, they indirectly alter the resident microbiota through metabolic interactions or by interacting with host epithelial cells and the epithelial immune system [24]. However, probiotics have not been well studied in the literature in relation to cats. In this study, we investigated the effect of a multistrain probiotic containing *Saccharomyces boulardii* and *Pediococcus acidilactici* in the diet on the intestinal flora of healthy short-haired domestic cats. The presented results indicate that the colonization of beneficial bacteria promotes gut health by modulating the gut microbiota, reducing inflammatory conditions, improving microbiota-derived SCFA production, and improving antioxidant status.

*Saccharomyces boulardii* belongs to *Saccharomyces cerevisiae*, with strong probiotic properties and antipathogen abilities. Studies have determined that *Saccharomyces boulardii* plays a significant role in the prevention of antibiotic-associated diarrhea [25]. Mixtures of probiotics such as *Saccharomyces boulardii* and *Bifidobacterium* reduce the severity of *E. coli* diarrhea in rats [26]. *Saccharomyces boulardii* mediates proinflammatory responses through yeast-secreted factors and signaling molecules that control inflammation, thereby regulating gastrointestinal inflammation [27]. *Pediococcus acidilactici* has the remarkable characteristics of lactic acid bacteria and can colonize the intestinal tract. Its metabolite organic acids can participate in physiological responses, such as by improving the balance of intestinal flora, promoting digestion and absorption, and improving immune performance [17]. *Pediococcus acidilactici* isolated from the feces of unweaned puppies was fed to weaned puppies, and it was observed that *Pediococcus acidilactici* can promote digestion and improve serum antioxidant capacity, thereby restoring the puppies’ appetite and reducing transportation stress [28]. Adding *Pediococcus acidilactici* to the diet of weaned piglets can increase the number of intestinal lactic acid bacteria, reduce the severity of diarrhea [29], and improve intestinal health. Studies have determined that a single- or multistrain probiotics can regulate intestinal function and treat gastrointestinal diseases [30]. How to correctly select the type and combination of probiotic strains is the key to achieving such outcomes.

OTUs are designed to facilitate analytical phylogenetic or population genetics studies, classifying and distinguishing all sequences according to similarity levels. In this study, compared with the control group, the probiotic group had 81 unique OTUs. Alpha refers to the diversity of fecal microbial communities and represents the richness and uniformity of species within the sample. In this study, there was no significant difference in the alpha diversity between the control and probiotic groups, which is consistent with Lin’s study of dogs using a fermentation product of *Saccharomyces cerevisiae*, which showed no change in the fecal alpha diversity [31]. Minamoto observed that the bacterial diversity and microbial community of dogs with idiopathic inflammatory bowel disease (IBD) were significantly reduced compared to those of the healthy control group [32]. In this study, a multistrain probiotic did not change the diversity of the gut microbiome of short-haired domestic cats, suggesting that a multistrain probiotic can be used safely in short-haired domestic cats.

The results of principal coordinate analysis (PCoA) in this study indicated no change in the microbial community structure between the control and probiotic groups. The weighted UniFrac distance map shows that the weighted UniFrac distance between the cats in the probiotic group is significantly greater than that in the control group, indicating that the differences in species and abundance of microbial communities between the two groups are great, which may be the result of individual differences between short-haired domestic cats. In a previous study, when multiple synbiotic preparations were administered to healthy cats and dogs, there was no significant difference in fecal microflora; however, it led to an increase in the abundance of probiotics in the feces of healthy cats and dogs [33]. Rossi studied the effect of multistrain probiotic SLAB51TM on cats suffering from chronic constipation and Hirschsprung’s disease and observed that it could significantly improve their clinical condition, with a significant increase in *Lactobacillus* spp. and Bacteroidetes; however, there was no significant difference in the microbiota between healthy controls and constipated cats [34]. LEFSe differential analysis showed that the 16 types of bacteria of the phylum Firmicutes and Proteobacteria played an important role in the control group. The study determined that the abundance of Proteobacteria is related to the increase in *Escherichia coli*, which belongs to Proteobacteria, has adhesion ability, invades the body, produces toxins, stimulates inflammatory cytokines, and is closely related to gastrointestinal diseases [35,36]. *Pediococcus* and Bacillaceae played an important role in the probiotic group. Bacillaceae have strong probiotic properties, such as the activity of secreted enzymes, which can help the host digest nutritional compounds and facilitate intestinal colonization [37]. The significant increase in the probiotic group may be associated with the use of a multistrain probiotic containing *Pediococcus acidilactici*, which can produce bacteriocin from ribosomes, colonize the intestine, regulate the composition of the intestinal microbiota, improve the host immune response, and enhance intestinal barrier function [38,39]. Studies have determined that bacteriocin-producing *Pediococcus acidilactici* can relieve constipation in mice and regulate intestinal flora [40]. The microencapsulation of probiotic strains by lyophilization can maintain microbial viability in cats and improve *Lactobacillus* count, exerting a positive effect on cat gut microbiota [41]. The supplementation of *Lactobacillus acidophilus* in healthy cat diets resulted in an increase the number of *Lactobacillus* in their feces and a decrease in the number of *E. coli*, which can effectively improve the fecal quality parameters and intestinal health of healthy adult cats [42]. In this study, the addition of *Saccharomyces boulardii* and *Pediococcus acidilactici* to the diet of healthy cats did not change the fecal microbial community structure, which varied due to individual differences; however, it promoted the colonization and abundance of beneficial bacterial strains and may have a positive effect on the gut microbiota of healthy cats.

The presented results show that there were no significant differences at the phylum and genus levels of microorganisms between the control and probiotics groups, and the dominant intestinal microbiota of the two groups are Firmicutes, Bacteroidetes, Actinobacteria, etc. Previous studies have also shown that the dominant microbiota of dogs’ and cats’ intestinal microorganisms are Firmicutes and Bacteroidetes [43], and their abundance values differ according to individual differences. The treatment of dextran sulfate sodium (DSS)-induced rats with *Saccharomyces boulardii* resulted in a decrease in Firmicutes/Bacteroidetes [44], whereas colitis was associated with increased numbers of Firmicutes and decreased numbers of Bacteroidetes. Bacteroidetes are abundant in healthy microbiomes. A decreased microbial abundance of Bacteroidetes was observed in cats with chronic enteropathy (CE) [45]. Firmicutes are associated with the production of short-chain fatty acids and other metabolites [46,47]. The host gut microbiota produces metabolites such as short-chain fatty acids (SCFAs) through fermentation, which play an important role in host/pathogen interactions [48]. The most common SCFAs are acetic, propionic, and butyric acids, which are involved in immunomodulatory and anti-inflammatory responses [49]. In this study, the concentrations of butyric acid and total SCFAs in the probiotic group were significantly higher than those in the control group. Coincidentally, previous studies have shown that DSS-induced rats have significantly increased acetic, propionic, and butyric acid levels, as well as total SCFA production, following the addition of multistrain probiotics, with a tendency to restore normal rats’ short-chain fatty acid levels (unpublished). Butyric acid has the strongest anti-inflammatory effect among all SCFAs [50]. Studies have shown that SCFAs play a crucial role in alleviating intestinal dysbiosis and maintaining intestinal homeostasis [51]. The gut microbiota is closely linked to host gut health, and an imbalance in the microbiota may cause host dybiosis, as well as intestinal and systemic diseases [52]. A characteristic feature of gut microbial dysbiosis is a decrease in obligate anaerobic flora, such as Firmicutes and Bacteroidetes, whereas there is an increase in facultative anaerobic bacteria, such as Enterobacteriaceae and *Escherichia coli* [53]. This study shows that a multistrain probiotic can improve the intestinal immune regulation and anti-inflammatory ability of healthy cats by adjusting the proportion of microbial compositions and increasing the production of SCFAs. Paradoxically, the multistrain probiotic did not alter the feline gut microbial diversity and structure presented in this study, although SCFA production was increased. It has been suggested that this may be due to probiotics playing a more significant role in the gut functional level compared to gut microbial composition [33]. Not coincidentally, a previous study reported that *Lactobacillus helveticus* Bar13 and *B. longum* Bar33 intake in humans promoted a significant increase in acetate and valeric acid levels but did not alter the structure of the fecal microbiota [54]. Meanwhile, inulin or short-chain fructooligosaccharides were reported to alter the short-chain fatty acid content in dog feces and modestly alter fecal flora abundance [55]. Therefore, further studies on the effect of probiotics on gut microbial function are necessary.

The degree of intestinal inflammation can be manifested by neutrophil derivatives, such as fecal calprotectin (FC), myeloperoxidase (MPO), and matrix metalloproteinases (MMPs) in feces [56]. Fecal calprotectin has an antibacterial effect and is proportional to the severity of inflammation [57]. Myeloperoxidase is known to catalyze the production of hypochlorous acid and promote the production of reactive oxygen species (ROS) and reactive nitrogen species (RNS), mediating oxidative stress and inflammation [58]. Matrix metalloproteinase-9 also activates underlying inflammatory molecules to modulate the inflammatory response [59]. Compared with the control group, the concentration of calprotectin and myeloperoxidase was lower in the probiotic group. Interestingly, Peptostreptococcaceae and Peptostreptococcales_Tissierellales, which are microbiota associated with inflammation, were relatively more abundant in the control group [60]. The content of MPO and FC of inflammatory markers in the probiotic group was significantly reduced, indicating that the addition of probiotics can regulate the intestinal inflammation-related flora and induce a decrease in the level of inflammatory markers, thereby reducing the inflammatory response. In our previous study, we observed that in DSS-induced rats, Peptostreptococcales and Peptostreptococcales_Tissierellales were relatively abundant, with the highest levels of inflammatory markers MPO, MMP-9, and FC, suggesting a relationship between inflammatory markers and inflammation-related gut flora (unpublished). Moreover, the antioxidant activity of superoxide dismutase (SOD) and glutathione (GSH) in the probiotic group was significantly increased. Studies have shown that the probiotic mixture affects the production of SCFAs in aging mice, and SCFAs may regulate antioxidant enzymes by inducing the expression of Nrf2 or HO-1, thereby directing antioxidant effects [12]. Studies have determined that probiotic-treated fermented sausages can not only reduce human inflammatory markers but also improve antioxidant plasma markers and increase butyric acid production [61]. The administration of *Saccharomyces boulardii*, which are associated with inflammation, has been observed to reduce FC levels in healthy adult dogs and maintain the stability of the intestinal environment [62]. *Lactobacillus* fermentum reduced the production of MDA and MPO and inhibited the release of proinflammatory cytokine tumor necrosis factor-α (TNF-α), IFN-γ, IL-1β, IL-6, and IL-12, effectively alleviating the symptoms of dextran sulfate sodium (DSS)-induced rats [63]. In a previous study, we observed that treatment administered with a multistrain probiotic in DSS-induced rats significantly reduced the levels of fecal inflammatory markers MPO, MMP-9, and FC; inhibited the activation of proinflammatory cytokines IL-6, IL-1β, interferon IFN-γ, and tumor necrosis factor TNF-α; increased the levels of antioxidant stress parameters SOD and GSH; and reduced the inflammatory state of rats (unpublished), consistent with the results obtained in this study. Following multistrain probiotic treatment, in this study, we observed that the metabolites of SCFA production may play a crucial role in the anti-inflammatory and antioxidant effects of cats.

## 5. Conclusions

In the present study, feeding a multistrain probiotic to healthy short-haired domestic cats did not alter their gut microbial structure and diversity. In addition, it promoted the colonization of beneficial bacteria (*Pediococcus* and Bacillaceae) and decreased the proportion of Firmicutes/Bacteroidetes. It was observed that the production of butyric acid and total SCFAs in feces increased, which was associated with Firmicutes and Bacteroidetes. Furthermore, SCFAs may have induced anti-inflammatory and antioxidant effects, not only reducing the concentration of fecal inflammatory markers MPO and FC but also increasing the activity of antioxidant enzymes SOD and GSH. Therefore, the presented results indicate that the colonization of beneficial bacteria promotes gut health by modulating gut microbes, improving microbiota-derived SCFA production, reducing the inflammatory state, and improving antioxidant status. Further studies on the effect of probiotics on the intestinal functional level (i.e., production of SCFAs) are needed. However, due to the influence of individual differences among cats and owner compliance, few studies have been conducted on cat gastrointestinal health in the literature. Importantly, it is necessary to explore the role of ideal probiotic strains in cat intestinal diseases to provide a theoretical basis to improve cat intestinal health.

## Figures and Tables

**Table 1:** Relative abundance of major flora at the phylum level (%).

Phylum	Control	Probiotics
Bacteroidota	12.30 ± 8.43	25.08 ± 19.68
Firmicutes	78.07 ± 13.66	53.93 ± 22.14

Data are presented as mean ± SD. There was no significant difference between the control and probiotic groups (*p* > 0.05).

**Table 2:** Statistical analysis of microbial alpha diversity.

Group	Chao	ACE	Shannon	Simpson	Evenness
Control	316.68 ± 27.52	315.58 ± 29.33	3.95 ± 0.25	0.05 ± 0.02	0.70 ± 0.03
Probiotics	284.35 ± 71.53	283.44 ± 70.47	3.55 ± 0.41	0.07 ± 0.03	0.64 ± 0.04

Data are presented as mean ± SD. There was no significant difference between control and probiotic groups (*p* > 0.05).

## Data Availability

The data underlying this article will be shared upon reasonable request to the corresponding author. The data are not publicly available due to the probiotics product is under development and patent application.
